# Physicochemical Evaluation of Cushuro (*Nostoc sphaericum* Vaucher ex Bornet & Flahault) in the Region of Moquegua for Food Purposes

**DOI:** 10.3390/foods12101939

**Published:** 2023-05-10

**Authors:** Sheda Méndez-Ancca, Renzo Pepe-Victoriano, Hebert Hernán Soto Gonzales, Abel Walter Zambrano-Cabanillas, Olegario Marín-Machuca, José Carlos Zapata Rojas, Maribel Maquera Maquera, Rosmery Fernandez Huanca, Jorge González Aguilera, Alan Mario Zuffo, Rafael Felippe Ratke

**Affiliations:** 1 Area of Marine Biology and Aquaculture, Faculty of Renewable Natural Resources, Arturo Prat University, Arica 1000000, Chile; rpepev@unap.cl; 2 Master’s Program in Aquaculture, Mention in Aquaculture of Hydrobiological Resources, Mention in Aquaponics, Arturo Prat University, Arica 1000000, Chile; 3 National University of Moquegua (UNAM), Ilo 18601, Peru; hsotog@unam.edu.pe (H.H.S.G.); maquera9620@gmail.com (M.M.M.); rous.fernandez.24@hotmail.com (R.F.H.); 4 Faculty of Oceanography, Fisheries, Food Science and Aquaculture, Academic Departments of Aquaculture and Food Science, Universidad Nacional Federico Villarreal, Lima 15001, Peru; azambrano@unfv.edu.pe (A.W.Z.-C.); omarin@unfv.edu.pe (O.M.-M.); 5 Asociación Las Brisas, Ilo 18601, Peru; carlindustri@gmail.com; 6 Department of Agronomy, Universidad Estadual de Mato Grosso do Sul (UEMS), Cassilândia 79540-000, MS, Brazil; j51173@yahoo.com; 7 Department of Agronomy, State University of Maranhão, Campus de Balsas, Balsas 65800-000, MA, Brazil; alan_zuffo@hotmail.com; 8 Department of Agronomy, Universidade Federal de Mato Grosso do Sul, Chapadão do Sul 79650-000, MS, Brazil

**Keywords:** calcium, iron, moisture, fiber, texture, nutritional value, correlations

## Abstract

The cyanobacterium *Nostoc* sp. contains considerable amounts of protein, iron, and calcium that could mitigate the problems of anemia and malnutrition in humans. However, the nutritional value of the edible species *Nostoc sphaericum* Vaucher ex Bornet & Flahault, which grows in the Moquegua region, is unknown. Descriptive research was developed, and samples were obtained from the community of Aruntaya, located in the region of Moquegua. Water samples were taken at two different points (spring and reservoir), and samples of the cyanobacteria were taken in the reservoir. The design used was completely randomized, with three repetitions. Sixteen characteristics associated with the water collected at two points were evaluated, and from the nutritional point of view, seven characteristics were evaluated in the collected algae. The physicochemical characteristics were determined using methods established in the Codex Alimentarius. For the morphological characterization at the macroscopic level, it was observed that the seaweed collected was spherical in shape, grayish-green in color, soft to the touch, and palatable. After carrying out the physicochemical and morphological characterization of the collected samples, it was verified that all were of *N. sphaericum*. When comparing the sixteen characteristics related to water at the two collection sites, highly significant differences (*p* < 0.01) were observed for most of the variables evaluated. The average data of the characteristics of the algae showed protein values of 28.18 ± 0.33%, carbohydrates of 62.07 ± 0.69%, fat of 0.71 ± 0.02%, fiber of 0.91 ± 0.02%, ash of 7.68 ± 0.10%, and moisture of 0.22 ± 0.01%. Likewise, calcium reported an average value of 377.80 ± 1.43 mg/100 g and iron of 4.76 ± 0.08 mg/100 g. High correlations (positive and negative) were obtained by evaluating seven characteristics associated with the reservoir water where the algae grew in relation to eight nutritional characteristics of the algae. In relation to the nutritional value, the amounts of protein, iron, and calcium exceed the main foods of daily intake. Therefore, it could be considered a nutritious food to combat anemia and malnutrition.

## 1. Introduction

In Peru, there is growing concern about anemia in the country’s public health policies [1]. Anemia directly affects children between 6 and 35 months of age, with a national average incidence of 38.8%, with values of 48.7% in rural areas and 35.3% in urban areas [2]. At the country level, the highest prevalences of anemia were recorded in Puno (69.9%), Cusco (57.4%), Huancavelica (54.2%), Ucayali (53.7%), Loreto (53.0%), Junín (52.6%), Madre de Dios (51.4%), and Pasco (50.2%) [3]. This scenario compromises the cognitive, social, and emotional development of infants, as reported by INEI [2] and Zavaleta and Astete-Robilliard [4].

One of the alternatives to combat anemia is the search for alternative foods of high nutritional value, taking advantage of the diverse natural resources that the country possesses [5]. *Nostoc* cyanobacteria, whose protein content is 25.4 g, 1076 mg of calcium, and 19.6 mg of iron per 100 g of *Nostoc* on a dry basis, constitutes an excellent alternative [6]. In dehydrated *Nostoc sphaericum* Vaucher ex Bornet & Flahault, known as “cushuro”, similar values were obtained by Neyra [7] when quantifying iron (15.72 ± 2.07 mg/100 g) and calcium (1260.13 ± 35.80 mg Ca/100 g), showing the nutritional value of this food.

*Nostoc* is a colony of cyanobacteria, when they are hydrated they can form spheres of 10 to 25 mm in diameter, similar to grapes. It is found in the Andean foothills above 3000 m [8]. In South America it is also known as cushuro, murmunta, llullucha, or llayta, known from Central America to Brazil, being consumed mainly in Peru and Bolivia. It exists in Asia, including China, Indonesia, and India, and in Europe, including Germany, Spain, and France. It is found in South Africa and used as a natural fertilizer [9]. In the mountains, it grows wild near rivers, lakes, and lagoons in the interior of the country, especially in the Puna region [10].

Studies by Celis-Plá et al. [11], Rosales-Loaiza et al. [5], Ponce [6], and Chili-Rodriguez and Terrazas-Viza [12] show that different species of freshly harvested cushuro contain from 35 to 42% proteins. According to Alegre et al. [13], the carbohydrate values reached 62.07 ± 0.69%; the same authors found values of 0.21 ± 0.03% fat, while Rosales-Loaiza et al. [5] reported a maximum value of 5.22 ± 1.74%. In addition, Macário et al. [14] found ω-3 and ω-6 fatty acids and minerals (Ca, P, Fe, Na, K), as well as all the essential amino acids and vitamins B1, B2, B5, and B8 [15]. The cyanobacteria contain bioactive compounds such as phycobiliproteins (especially C-phycocyanin) and methyl palmitate (C16:0) [16]; *Nostoc* sp. contains polyphenols, phycocyanin, and ascorbic acid [11,17,18]. These properties would help to improve the nutritional status of people who consume it, in combating anemia and improving the nutritional status of children between one and three years of age [19].

Numerous studies, at the level of microalgae, have been described and show the importance of cyanobacteria [14,15,20]. In contrast, there is little information related to the exploitation of macroalgae on a large scale, so research that contributes to the knowledge of the living conditions and nutritional composition of the freshwater algae *Nostoc* sp. is necessary [20]. In a region where this type of algae is abundant, it would be of great relevance to obtain information on this species of freshwater algae [10,21,22]. Knowing the nutritional characteristics and the basis for establishing cultivation parameters for small- and large-scale production can contribute to eradicating problems such as anemia and malnutrition, using *Nostoc* sp. from the water resources of the Moquegua Region.

The objective of this study was to determine the water quality conditions under which the macroalga *N. sphaericum* grows naturally, by analyzing the physical and chemical parameters of the water in the growth area; likewise, the proximal composition of the macroalgae was analyzed on a dry basis. Both objectives were developed to determine the nutritional value of freshwater algae.

## 2. Materials and Methods

### 2.1. Collection Site

**Figure 1:**
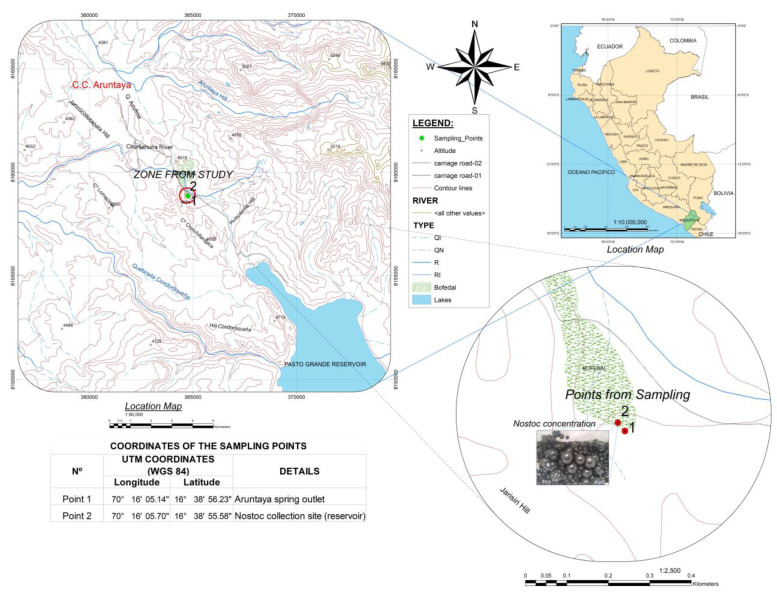
Representation of the collection site of the cyanobacteria studied.

*Nostoc* algae samples were collected from streams (16°36′19.9″ S, 70°18′1.2″ W) located in the Aruntaya farming community at an altitude of 4434 masl in the Carumas district, Mariscal Nieto Province, Moquegua region (Figure 1).

**Figure 2:**
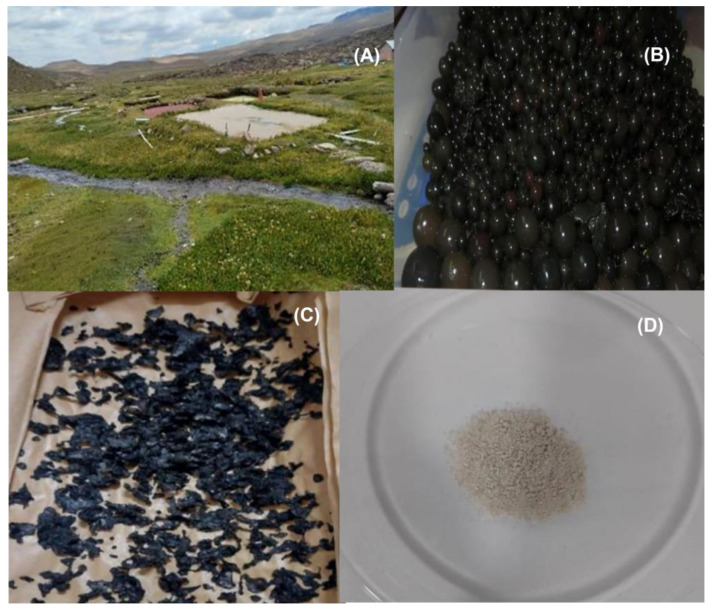
This collection site (**A**), sample of collected cyanobacteria (**B**), subsequently dried (**C**) and macerated (**D**).

The temperature of the water “in situ“ was recorded with a basket thermometer. Subsequently, the collected biological sample (water and cyanobacteria) was transported in an ice cooler of 10 L capacity, inside which 6 gel pack bags of 0.5 L were placed to keep the temperature below 5 °C. The *Nostoc* was protected with reusable wet cloths of size 33.5 × 34.2 cm to keep them moist (Figure 2B), inserting a layer of damp cloth and another layer of *Nostoc* until completing the total volume of the cooler. This sampling protocol allowed the samples to be transferred to distant places in a reduced space under adequate conditions for the conservation of the biological sample. The collection method of all the experimental samples was developed in the present work.

Under laboratory conditions, the algae were initially identified as *N. sphaericum* (Figure 2B), according to the classification key described by Bornet and Flahault [23], which is integrated into the classification system [24]. For the identification of *Nostoc sphaericum*, the Geitler taxonomic key [25] and the Guiry classification system [26] were used.

### 2.2. Physical and Chemical Analysis of the Water Where the Collection Took Place

Water samples (1000 mL) were collected at the Aruntaya spring and 20 m from it (reservoir), all in triplicate. In the growth zone of the *N. sphaericum* strain, three samples were collected and sent to the LABINVSERV Research and Services Laboratory, which used the AOAC method [27] for pH analysis and the gravimetric method for dissolved solids and total hardness. Alkalinity and acidity were determined by the volumetric method, and sulfates were determined by the AOAC [27] turbidimetric method. Phosphates, ammonia nitrogen, total nitrogen, BOD5, and COD were determined by the AWWA method [28]. Likewise, the Certificaciones del Perú S.A. CERPER Testing Laboratory determined the amount of nitrites and nitrates by the anion method by ion chromatography: EPA Method 300.0 1993 [29].

### 2.3. Physical Analysis of Samples

The physical characteristics were estimated from three fresh samples of *N. sphaericum*. The moist matter moisture was estimated by protocol 930.04 [30]. The dry matter moisture of 1 kg of *N. sphaericum* (Figure 2C) was characterized by the hot air oven method at 70 °C for 5 h [27]. According to the method of Torres-Maza et al. [31], the hydrocolloids were dried in a LABCONCO freeze dryer (Models 79480, Labconco Corp., Kansas City, MO, USA) at a pressure of 0.004 bar, with an initial temperature of −40 °C, and an air dryer (CORP. JARCON model SBT—10 × 10) at a constant temperature of 40 ± 0.5 °C, with a velocity or flow rate of 20 m^3^ min^−1^ [5], to determine the humidity in an air oven.

The color was determined by the spectrophotometric test method, through which the chromatic coordinates were determined, which is determined by the CIE L*a*b* model [32], and texture was determined by the sensory test method.

Cushuro samples were sent to the testing and research laboratory “Sistema de Servicios y Análisis Químicos S.A.C. (SLAB) to determine the proximate content of *N. sphaericum*. Proteins were determined according to the Kjeldahl method, crude fat content was obtained by the Soxhlet method, and carbohydrates, total ash, and fiber were determined according to the official method of the AOAC [27]. Finally, the amounts of iron and calcium were determined by atomic absorption, according to protocol 975.03 [30].

### 2.4. Experimental Design and Statistical Analysis

A completely randomized experimental design was used, with two treatments associated with algal tail points. Normality and homogeneity of variance were tested using the Kolmogorov–Smirnov test. Subsequently, analysis of variance (ANOVA) was performed, and Tukey’s multiple range comparison test (*p* < 0.01) was used to determine differences between treatments. The experimental data were processed using the RBio program [33].

## 3. Results

### 3.1. Physicochemical Analysis of Water

When verifying the results of the ANOVA (Table 1), it was observed that when comparing the two points of the algal tail, highly significant differences (*p* < 0.01) were obtained for most of the variables, with the exception of pH, which did not show differences (Table 1). Alkalinity, total hardness, nitrites as N, and nitrites as NO_2_ did not show variations in the data collection, and for this reason, they were included in the analyses in Table 1. Under these conditions, the coefficients of variation were low, showing the precision of the data obtained for all the variables evaluated (Table 1).

When the comparisons of means were carried out by Tukey’s test, the results are shown in Table 2. The results show the water quality values of the Aruntaya spring water coming from the subsoil at a temperature of 11.7 °C on average, which at 20 m distance from its origin (Figure 1) feeds the lentic body (or reservoir) where the strain of *N. sphaericum* was collected, and the water is at an average temperature of 10.7 °C, demonstrating a decrease in the temperature of the water during its journey to the reservoir.

The water quality analysis showed that the water from the Aruntaya spring had the highest amount of sulfates, 2 mg L^−1^, phosphates 0.36 mg L^−1^, and nitrates 0.622 mg L^−1^. Likewise, it can also be observed in Table 2 that in the reservoir, the biochemical oxygen demand (BOD5) increased by 7.00 mg L O_2_^−1^, and the chemical oxygen demand (COD) increased by 22,955 mg L O_2_^−1^; similarly, ammonia nitrogen reached values of 0.15 mg L^−1,^ and total nitrogen reached 1.58 mg L^−1^. In the same way, increases are observed in pH, 0.1; conductivity, 1.1 µS cm^−1^; dissolved solids, 1.1 mg L^−1^; alkalinity (permanence invariable); and acidity, slightly increased by 0.09 mg L^−1^ (Table 2). On the other hand, the water from the area where the cyanobacterium *N. sphaericum* grows presented lower values of sulfates (21.40 mg L^−1)^, phosphates (0.20 mg L^−1)^, and nitrates (NO_3_) (0.04 mg L^−1)^, as shown in Table 2. Another outstanding characteristic of the water quality of the *N. sphaericum* collection area (growth area) is the low amount of Nitrospira (NO_2_), which is less than 0.004 mg L^−1^.

### 3.2. Physical Characteristics of N. sphaericum

After analyzing the cushuro samples at the Certified Laboratory of Sistema de Servicios y Análisis Químicos S.A.C., it was found that the fresh sample was *N. sphaericum*, which had an average fresh moisture content of 98.73%, while the dry sample had an average moisture content of 0.222 ± 0.005% in 100 g of dry matter. The chromatic coordinates of the CIE L*a*b* model showed color values of 92-9-89, on average, which corresponds to a grayish-green color. In terms of texture, the fresh samples were soft to touch and spherical in shape (Figure 2B).

### 3.3. Chemical Characteristics of N. sphaericum

Table 3 reports the chemical characteristics of the analyzed samples of *N. sphaericum,* whose average values are as follows: protein 28.18 ± 0.33%, carbohydrates 62.07 ± 0.69%, fat 0.71 ± 0.02%, fiber 0.91 ± 0.02%, ash 7.68 ± 0.10%, and moisture 0.22 ± 0.01%. Likewise, calcium had an average value of 377.80 ± 1.43 mg/100 g, and iron had an average value of 4.76 ± 0.08 mg/100 g. The chemical characteristics of the collected sample are compared with other values for the same species of cyanobacteria, described in previous studies (Table 3).

### 3.4. Correlations Established between Water and Chemical Characteristics of N. sphaericum

**Figure 3:**
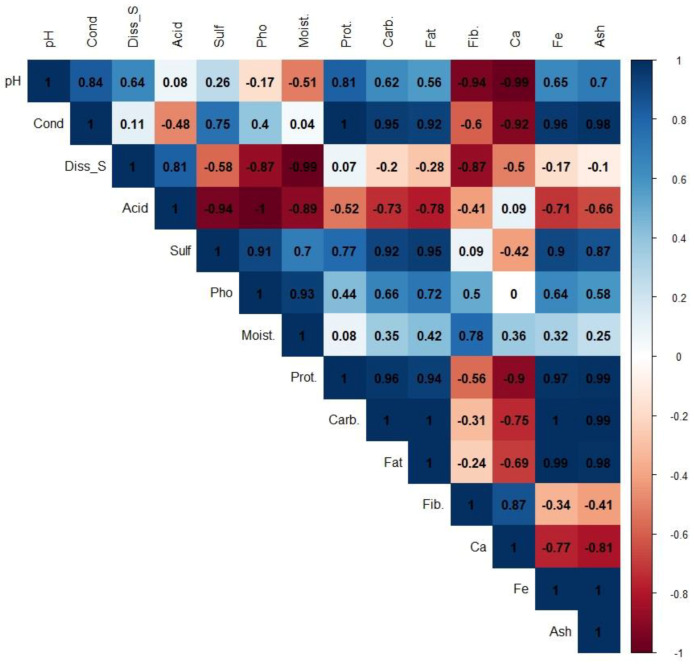
Pearson’s correlation was obtained by associating the characteristics obtained for the water quality of reservoirs and the nutritional properties of cyanobacteria. pH: pH of water, Cond: conductivity, Diss_S: dissolved solids, Acid: acidity, Sulf: sulfates, Pho: phosphates, Moist: moisture, Prot: proteins, Carb: carbohydrates, Fat: fat, Fib.: fiber, Ca: calcium, Fe: iron, and Ash: ash.

To determine the degree of correlation that exists between the variables obtained by evaluating the reservoir water and the properties of the algae produced under these conditions, an analysis of correlations between these variables was established, and a matrix of correlations was built, which is shown in Figure 3.

When evaluated, the matrix correlations are observed to be of moderate to high magnitude in relation to the pH of the pond water. The pH showed positive correlations with the protein content (0.81) and negative correlations with the fiber (−0.94) and calcium (−0.99) contents. The conductivity of the water was the characteristic that was most associated with the characteristics linked to the nutritional quality of the cyanobacteria (protein (1), carbohydrates (0.95), fat (0.92), calcium (−0.92), iron (0.96), and ash (0.98)) (Figure 3). The acidity of the water was negatively correlated with the content of protein (−0.52), carbohydrates (−0.73), fat (0.78), iron (−0.71), and ash (−0.66). The concentration of sulfates in the water was positively correlated with the content of protein (0.77), carbohydrates (0.92), fat (0.95), iron (0.90), and ash (0.87). The concentration of phosphates in the water showed positive correlations, but of lesser magnitudes in relation to those found with sulfates, with values of 0.44 for protein content, 0.66 for carbohydrates, 0.72 for fat, 0.5 for fiber content, 0.64 for iron, and 0.58 for ash (Figure 3). The correlations obtained indicate that there are physical parameters of the water that determine the nutritional behavior of the bacteria for the evaluated conditions.

## 4. Discussion

Among the most globally distributed algae, we can find *N. commune* [36]. *N. sphaeroides* is another of the most common algae used as a dietary macroalgae for its nutritional value [37,38]. In this study, we verified the distribution of macroalgae of *N. sphaericum* in the Moquegua region and how water quality influences its growth and nutritional value.

### 4.1. Quality of the Water Where Sampling Was Performed

The main place where most algae develop naturally is in riverbeds. Knowing the quality of the water where they develop is important to establish cultivation parameters for this species. The water of the Aruntaya spring has a high amount of chemical compounds (22 mg L^−1^ of sulfates, 0.36 mg L^−1^ of phosphates, and 0.622 mg L^−1^ of nitrates), which are nutrients used to feed *N. sphaericum*. These compounds were correlated with nutritional contents of the evaluated algae, which shows the possibility of obtaining these algae in the characterized reservoir, and hence its potential use.

When we looked at the values for the sampling zone, the cyanobacteria growth area, the nutrients were significantly reduced, to values of 21.40 mg L^−1^ sulfates, 0.20 mg L^−1^ phosphates, and 0.04 mg L^−1^ nitrates. This result indicates that these components were absorbed by the cyanobacteria and contributed to their development, as well as that the amounts present in the water allowed adequate development of the algae under these conditions. This result corresponds to that described by Silambarasan et al. [39], who mentioned that *Nostoc* sp. remove TP (total phosphorus), NH_4_ (ammonium), and TN (total nitrogen) from 6 to 10 days of culture, while COD (chemical oxygen demand) decreases in 10 days. The results of this research also agree with those described by Nagappan et al. [40], when expressing that *Nostoc* sp. is a nitrogen-fixing cyanobacterium. Khan et al. [41] found that *Nostoc* sp. assimilates NH_4_-N more efficiently and can integrate it in the form of amino acids, which are important for cyanobacterial growth [42]. The results show that these parameters are altered as a result of the absorption and growth capacity of the cyanobacteria, a fact confirmed by Silambarasan et al. [39]. These results also confirm that the reservoir water where the collected cyanobacteria grow has adequate characteristics to facilitate the growth of the algae.

In the sample collection area, the biochemical oxygen demand (BOD5) increased by 7.00 mg L O_2_^−1^, similar to the chemical oxygen demand (COD), which increased by 22.955 mg L O_2_^−1^. Variations in these parameters, according to Makki and Khudhair [43] and Soto et al. [44], mean that the organic material has increased, therefore, there is greater oxygen consumption. Similarly, ammonia nitrogen reached values of 0.15 mg L^−1,^ and total nitrogen reached 1.58 mg L^−1^, which were higher in the collection area due to the greater amount of organic material in the collection area. Another outstanding characteristic of the water quality of the *N. sphaericum* collection area is the low amount of Nitrospira (NO_2_), which is less than 0.004 mg L^−1^, permissible to achieve the growth of *N. sphaericum* [45]. From the precedents, it can be deduced that Aruntaya spring water contains the nutrients necessary for the growth of *Nostoc* sp. (Table 1).

### 4.2. Physical Characteristics of N. sphaericum

The fresh sample of *N. sphaericum* exhibited a grayish-green color, a soft texture to the touch, and a spherical shape, characteristics that confer a pleasant appearance, so it could possibly have a high degree of acceptability to the consumer. It is also known that species such as *N. flagelliforme* have been consumed in China [46] and *N. commune* in Peru [47] since pre-Columbian times [15], and the cyanobacterium *N. sphaericum* is consumed in different “gourmet” dishes internationally [48]. The values of chromatic coordinates mentioned for *N. sphaericum* (L* = 92, a* = −9, −14.6, b* = −89) indicate a very bright yellow-green color. If we compare these values with other colors common in nature, we could say that it resembles a color such as that of certain varieties of Golden Delicious Malus apples [49] and the dark greenish color of the nori macroalgae [50] used in making sushi. As shown by Li et al. [51], who measured the chromatic coordinates of the Golden Delicious apple, the chromatic coordinates for the fresh apple were L* = 92.46, a* = −2.55, and b* = 26.25. Likewise, Olguin-Santana and Jacobo-Velázquez [52] indicated the chromatic coordinates of fresh nori seaweed at different times of its culture. The average values reported were L* = 35.6 to 47.2, a* = −10.3 to −14.6, and b* = −11.6 to −18.6, verifying the color similarity between them.

### 4.3. Chemical Characteristics of N. sphaericum

It has been described that *N. sphaericum* is characterized by presenting molecules of high nutritional value (Table 3), such as proteins, that reach percentages of 31.23 ± 3.07% [5], calcium of 145 ± 8.80 mg/100 g [34], iron of 19.60 mg/100 g, and phosphorus of 258 mg/100 g [6]. The values reported in the present investigation were similar (protein, 28.18 ± 0.33%; carbohydrates, 62.07 ± 0.69%; fat, 0.71 ± 0.02%; fiber, 0.91 ± 0.02%; ash, 7.68 ± 0.10%; and moisture, 0.22 ± 0.01%) and show the possibility of collecting algae with excellent nutritional properties. Likewise, calcium had an average value of 377.80 ± 1.43 mg/100 g, and iron had an average value of 4.76 ± 0.08 mg/100 g. The values obtained in this study show that this cyanobacterium has high nutrient contents and that these values are highly correlated with variables associated with pond water, which means that as a whole it is possible to be used a food that can contribute to daily intake.

The protein content of *Nostoc* sp. in dry matter varies according to the type of strain; for example, Celis-Plá et al. [11] reported a value of 47.71 ± 0.30 for *N. calcicola*, Rosales-Loaiza et al. [5] reported a content of 31.23 ± 3.07% for the *Nostoc* LAUN0015 strain, and for Ponce [6], *Nostoc* reached a value of 25.40%. Finally, Chili-Rodriguez and Terrazas-Viza [12] reported 20.00%, and in the research developed, *N. sphaericum* reached a similar protein content, equal to 28.18 ± 0.33%, surpassing in quantity the animal origin proteins contained in milk, quinoa, egg, meat, and fish, containing only 3.29%, 16.30%, 16.40%, 20.10%, and 23.1%, respectively [45]. *N. sphaericum* has all the essential amino acids, as shown by Corpus-Gomez et al. [15], while containing easily digestible proteins of plant origin [53].

The carbohydrate values for *N. sphaericum* in the investigation were 62.07 ± 0.69%, predominant compared to the other macromolecules that form the cyanobacteria. Similar results, of 46.40 ± 0.59%, 62.4%, and 74.2% were found by Rosales-Loaiza et al. [5], Ponce [6], and Chili-Rodriguez and Terrazas-Viza [12], respectively. In relation to the degree of digestibility of carbohydrates, according to Capcha et al. [53], carbohydrates are rapidly digestible because they do not contain cellulose. Another experimental study, by Li et al. [54], used *N. commune* Vauch. polysaccharide fermented by *Lactobacillus* for therapeutic treatment of rats, achieving a reparative effect on Cd-induced renal injury, inhibiting apoptosis, and improving the composition of the intestinal microbiota. In addition, according to Guo and Li [55], it inhibits the occurrence of colon tumors in mice. Fiber, in this study, presented values ranging from 0.90% to 1.64 ± 0.57% [5,12,13], which is important for the digestion of short-chain fatty acids [56].

In this work, lipids in *N. sphaericum* were 0.71 ± 0.02%; similarly, Alegre et al. [13], for the same species, found a lower percentage of lipids, of 0.21 ± 0.03%. Macário et al. [14] highlighted that *N. muscorum* lipids contain important polyunsaturated fatty acids such as ω-3 and ω-6, which have anti-inflammatory and cardiovascular disease prevention properties for humans [14,57,58].

Likewise, in this investigation, the fiber content of *N. sphaericum* was 0.91 ± 0.02%; for the same species, Torres-Maza et al. [31] reported a content of 0.03 ± 0.07%, lower than the value found in this study. However, for Alegre et al. [13], the crude fiber content for *Nostoc* sp. was higher, reaching a value of 5.77 ± 0.11% of crude fiber. This difference in fiber content is probably due to the different environmental conditions of growth of the strain and the drying method, which were different for each investigation, and humidity reached in the dehydration of the matter. According to Castro et al. [58], the drying method and the applied temperature are inversely proportional, and they concluded that for the infrared drying of macroalgae, a temperature of 40 °C should be considered, to avoid the degradation of color and algae components.

There is also variability in the ash content. In the investigation, a value of 7.68 ± 0.10% was obtained, while in other studies, Alegre et al. [13], for the *N. sphaericum* strain, and Rosales-Loaiza et al. [5], for *Nostoc* LAUN0015, reported values of 7.77 ± 0.01% and 19.33 ± 2.76%, respectively, of ash. However, Torres-Maza et al. [31] found a value of 0.13 ± 0.01% ash for the *N. sphaericum* species, which is a low content and similar to most fruits and vegetables, that have an ash content between 0.3 and 1%. In general, ash accounts for less than 5% of the dry matter of food. Thus, the result obtained in the investigation is similar to the amount of ash present in vegetables [59], and shows the potential of the cyanobacteria collected under the conditions of Moquegua, Perú.

In relation to calcium, in dry matter, Alegre et al. [13] obtained 1224.4 mg Ca/100 g in cushuro from *Nostoc* sp., while this research reported a lower value, of 377.80 ± 1.43 mg Ca/100 g. However, the amount of calcium is higher in relation to milk, quinoa, egg, meat, and fish, containing 32, 27, 56, 12 and 28 mg Ca/100 g of food, respectively [45]. Regarding iron, Ponce [6] found 19.6 mg Fe/100 g in *Nostoc* sp., while *N. sphericum* in the present investigation contained 4.76 ± 0.08 mg Fe/100 g of dry matter, higher than milk quinoa, egg, meat, and fish, which only contain 1.3, 0.8, 1.1, 2.3, and 1.2 mg Fe/100 g of feed, respectively [45].

Iron is very important to mitigate anemia, and, therefore, its antecedents are addressed with greater emphasis. It is highlighted that the recommended daily amount of consumption is 8 mg of iron for men and 18 mg for women. Beef contains 2.6–3.0 mg/100 g, while liver contains 18.6 mg/100 g, legumes such as beans and lentils contain 1–2 mg/100 g, and spinach contains approximately 3.6 mg of iron/100 g [60]. On the other hand, clams are the richest source of iron at 28 mg/100 g [61], and Bhutan red rice contains approximately 7.6 mg/100 g [62]. It has been shown that iron absorption can be improved by consuming foods rich in vitamin C together with foods rich in iron [63].

Fleurence et al. [64] found that some species of macroalgae can contain up to 4.4 mg/100 g of dry weight. Another study, by Ganesan and Kumar [65], evaluated the effect of an extract of the macroalgae red seaweed *Gracilaria corticata* on iron deficiency anemia in rats, and the results showed that the administration of the extract significantly improved hemoglobin levels. In the investigation, it was found that the macroalga *N. sphaericum* reached a value of 4.76 ± 0.08 mg Fe/100 g of dry matter. Therefore, it can have effects on the prevention and treatment of iron deficiency. However, more studies are needed to determine the efficacy of macroalgae and their extracts as dietary supplements in humans.

One of the outstanding results of this research is the nutrient content of *N. sphaericum*, with values above the average of other important foods in the diet of most humans. These results suggest that this species of cyanobacteria could be considered a food of high nutritional value [12,53]. Research with a focus on modern medicine showed that *N. sphaeroides* contains a variety of essential amino acids, polysaccharides, and other bioactive substances [36], that highlights its nutritional value. Likewise, Celis-Plá et al. [11], Xu et al. [17], and Li et al. [18] concluded that *N. calcícola* and *N. sphaeroides* contain polyphenols, phycocyanin, and ascorbic acid, that have antioxidant, anti-inflammatory, and anti-infective therapeutic effects, respectively.

Another study has shown that the microalga Nostoc LAUN0015, grown with nitrogen injection, produces large amounts of biomass, reaching values of 890 μg mL^−1^ [5], evidencing an alternative for large-scale production; however, the authors of [5,65,66,67,68] highlight the potential of cyanobacteria as a source of food and nutrients, as well as their importance in the fight against malnutrition and food security. These authors also point out the importance of addressing the challenges associated with the sustainable cultivation and production of these microalgae to maximize their positive impact on society and the environment. It is important to highlight that, until now, most of the studies have focused on production at the microalgal level, leaving the focus on the mass production of the species pending.

Consequently, the results obtained in the present research, and the previous accounts on water quality and proximal composition of the macroalga *N. sphaericum,* have promising possibilities of application, e.g., in pharmaceutical industries and possible applications in biotechnology. The verified nutritional value confirms that this cyanobacterium can contribute to the prevention of anemia, mitigate malnutrition due to food deficits, and serve as a potential food for humans and animals.

## 5. Conclusions

In conclusion, the study has managed to determine the water quality conditions necessary for the natural growth of the macroalgae *N. sphaericum*, which will improve the production and use of this natural resource.

On the other hand, the macroalgae *N. sphaericum* is a rich source of nutrients, reaching a protein content of 28.18 ± 0.33%, carbohydrates of 62.07 ± 0.69%, fat of 0.71 ± 0.02%, fiber of 0.91 ± 0.02%, ash of 7.68 ± 0.10%, moisture of 0.22 ± 0.01%, calcium of 377.80 ± 1.43 mg/100 g and iron of 4.76 ± 0.08 mg/100 g, values that demonstrate the potential of macroalgae as food for humans and animals.

## Figures and Tables

**Table 1:** ANOVA results obtained when evaluating the physicochemical analysis of water from *N. sphaericum* collected in the Carumas district, Mariscal Nieto Province, Moquegua region.

Analysis	*p* Value	Significance	CV (%)
pH	7.342	0.0536	2.13
Conductivity (µS cm^−1^)	697.7	1.22 × 10^−5^	0.06
Dissolved solids (mg L^−1^)	180,000	1.85 × 10^−10^	0.01
Acidity (mg L^−1^)	14.31	0.0194	0.81
Sulfates (mg L^−1^)	20.55	0.0106	0.93
Phosphates (mg L^−1^)	867	7.92 × 10^−6^	2.57
Ammonia nitrogen (mg L^−1^)	2.804 × 10^32^	<2 × 10^−16^	1.46
Total nitrogen (mg L^−1^)	1.215 × 10^32^	<2 × 10^−16^	6.05
BOD5 (mg L O_2_^−1^)	8.769 × 10^30^	<2 × 10^−16^	6.36
COD (mg L O_2_^−1^)	1.482 × 10^30^	<2 × 10^−16^	1.01
Nitrates (as N) (*) (mg L^−1^)	1.239 × 10^31^	<2 × 10^−16^	6.03
Nitrates NO_3_ (*) (mg L^−1^)	1.268 × 10^31^	<2 × 10^−16^	6.05

Source: Laboratorio de Investigación y Servicios LABINVSERV-UNSA, (*) Laboratorio de Ensayo de Certificaciones del Perú S.A. CERPER. CV: coefficient of variation.

**Table 2:** Physicochemical analysis of water from *N. sphaericum* collected in the Carumas district, Mariscal Nieto Province, Moquegua region.

Analysis	Unit	Aruntaya Spring Water	*N. sphaericum* Growth Water (Reservoir)
pH		7.61	7.98 ^NS^
Conductivity	µS cm^−1^	89.99 b	91.20 a
Dissolved solids	mg L^−1^	53.00 b	55.00 a
Alkalinity	mg L^−1^ CaCO_3_	20.85	20.85 ^NS^
Acidity	mg L^−1^	6.30 b	6.46 a
Sulfates	mg L^−1^	21.96 a	21.21 b
Phosphates	mg L^−1^	0.36 a	0.19 b
Total hardness	mg L^−1^	30.32	30.32 ^NS^
Ammonia nitrogen	mg L^−1^	0.00 b	0.15 a
Total nitrogen	mg L^−1^	2.11 b	3.69 a
BOD5	mg L O_2_^−1^	42.00 b	49.00 a
COD	mg L O_2_^−1^	217.56 b	240.51 a
Nitrates (as N) (*)	mg L^−1^	0.140 a	0.01 b
Nitrates NO_3_ (*)	mg L^−1^	0.622 a	0.04 b
Nitrites (as N) (*)	mg L^−1^	<0.001	<0.001 ^NS^
Nitrites NO_2_ (*)	mg L^−1^	<0.004	<0.004 ^NS^

Source: Laboratorio de Investigación y Servicios LABINVSERV-UNSA, (*) Laboratorio de Ensayo de Certificaciones del Perú S.A. CERPER. Means with equal letters do not differ significantly in the same line (Tukey *p* < 0.01). ^NS^: no significant differences.

**Table 3:** Comparative table of chemical parameters of *Nostoc sphaericum*, Carumas district, Mariscal Nieto Province, Moquegua region.

Authors	Present Study	a ^1^	b		c	d		e		f		g
Strain Type	*N. sphaericum*	*Nostoc*	*N.* *sphaericum*		*Nostoc*	*N.* TISTR 8872	*N.* TISTR 8873	*N.* LAUN0015	*N.* UAM206	*N.* *commune*	*N.* *sphaericum*	*N.* *calcicola*
Dry weight								0.98 ± 0.10	0.75 ± 0.10			
% Protein	28.18 ± 0.33		20.00	0.60	25.40			31.23 ± 3.07	25.15 ± 1.56	1.21 ± 0.02	1.14 ± 0.03	47.71 ± 0.30
% Carbohydrates	62.07 ± 0.69		74.20	2.23	62.40	30.70	32.90	43.62 ± 3.59	46.40 ± 0.59	2.48 ± 0.03	2.26 ± 0.01	33.95 ± 6.66
% Fat	0.71 ± 0.02		0.30	0.01	0.80			1.05 ± 0.45	0.74 ± 0.49	0.31 ± 0.02	0.31 ± 0.00	5.22 ± 1.74
% Fiber	0.91 ± 0.02		0.90	0.03				1.64 ± 0.57	1.31 ± 0.76	0.02 ± 0.03	0.03 ± 0.07	
Calcium mg/100 g	377.80 ± 1.43	145 ± 8.80			1.08							
Iron mg/100 g	4.76 ± 0.08	0.75 ± 0.09			19.60							
Phosphorus					258.00							
% Ash	7.68 ± 0.10		4.60	0.14	5.10			19.33 ± 2.76	26.40 ± 3.40	0.20 ± 0.00	0.13 ± 0.01	
% Humidity	0.22 ± 0.01			97.00				11.73 ± 1.30	12.80 ± 1.79	95.77 ± 1.60	96.12 ± 1.22	
Observation	Dry matter	Dry matter	Dry matter	Wet matter	Dry matter	Dry matter		Dry matter		Wet matter		Dry matter

^1^ other articles (a) Obana et al. [34], (b) Chili-Rodriguez and Terrazas-Viza [12], (c) Ponce [6], (d) Sirajunnisa and Surendhiran [35], (e) Rosales-Loaiza et al. [5], (f) Torres-Maza et al. [31], (g) Celis-Plá et al. [11].

## Data Availability

Data supporting the findings of this research are available from the corresponding author upon request.
